# Design and Manufacturing Optoelectronic Sensors for the Measurement of Refractive Index Changes under Unknown Polarization State

**DOI:** 10.3390/s21217318

**Published:** 2021-11-03

**Authors:** Damian Harasim, Piotr Kisała, Bakhyt Yeraliyeva, Janusz Mroczka

**Affiliations:** 1 Department of Electronics and Information Technology, Lublin University of Technology, 20-618 Lublin, Poland; d.harasim@pollub.pl; 2 Department of Information Systems, M.Kh. Dulaty Taraz Regional University, Taraz 080000, Kazakhstan; b_eral@mail.ru; 3 Department of Electronic and Photonic Metrology, Wroclaw University of Technology, 50-317 Wroclaw, Poland; janusz.mroczka@pwr.wroc.pl

**Keywords:** photonic sensor, refractive index measurement, tilted Bragg gratings

## Abstract

This article proposes a new method for detecting slight refractive index changes under conditions of unknown polarization state. It is argued that an insignificant modification of the tilted fiber Bragg grating (TFBG) structure and selecting the appropriate spectral region allows us to accurately track changes in the refractive index. It has also been proven that the method can be easily made insensitive to temperature and that the sensitivity to changes in the polarization plane of the input light can be significantly reduced, which is crucial in later practical applications. Analytes in the form of an aqueous glucose solution were used to calibrate the sensor. The proposed method, based on perpendicular tilted fiber Bragg grating (P-TFBG), has a wide range of universality because its development and slight modification will enable the detection of glucose, pathogens, and viruses.

## 1. Introduction

Optical methods are becoming increasingly important in the detection of many physical and chemical quantities [1,2]. This is undoubtedly due to the dynamic advances in optical techniques [3,4,5], which include specialties such as spectroscopic and imaging techniques. Among many, the most promising seem to be high-resolution microscopy techniques [6,7], surface plasmon resonance [8,9,10], Fourier transform infrared spectroscopy [11], near-infrared spectroscopy [12], light emission caused by electronic relaxation techniques (fluorescence) [13,14,15,16], and Raman spectroscopy [17,18].

Advances in the miniaturization of light sources such as superluminescent diodes (SLEDs) or tunable lasers enable the construction of compact photonic measurement systems and the development of new measurement methods. Photonics-based tools are becoming increasingly popular in photonics, especially in the area of pathogen detection [19]. Very promising detection methods include the use of photonic periodic structures located in the core of an optical fiber. The simplest examples of such photonic structures are uniform Bragg gratings [20]. The first modified fiber Bragg grating (FBG) sensor that directly detects the refractive index changes in pathogen detection through patient saliva was proposed by Samavati et al. [21]. It is a highly sensitive FBG optical fiber sensor designed to detect the COVID-19 virus accurately with the capability to act as a remote device. However, this solution requires an additional Au nanolayer to be applied in the region where the FBG element is written. On the other hand, in 2020, F. Esposito et al. proposed a real-time method for the detection of the refractive index in virus detection using long period gratings (LPG) [22]. This solution requires LPG to be fabricated in a W-type double cladding optical fiber. To provide proper sensitivity, the outer cladding diameter of the fiber must be modified, which requires additional technological treatment. Udos et al. demonstrated a biosensor based on the surface plasmon resonance tilted fiber Bragg grating (SPR-TFBG) for the detection of the refractive index in order to detect enterovirus A71 [23]. In this case, it is required to cover the TFBG optical fiber with a layer of gold to obtain an SPR effect. This effect can also be caused by the use of metallic layers other than gold. In some cases, for resonances close to the guided mode cut off (i.e., the wavelength at which resonances are no longer guided by the cladding-surrounding medium interface), the S and P resonances of a normal TFBG overlap [24,25].

The polarization state of the input light affects the optical performance of TFBG structures. The twisting of the already tilted refractive index modulation causes a decrease in the sensitivity of such a structure to the rotation angle of the input light polarization. Optical fibers and conventional, homogeneous periodic fiber structures written on them are not sensitive to changes in the polarization of the input light because they are cylindrically symmetric. Such symmetry is their important advantage because there is no need to use polarized light when measuring signals coming from such structures (e.g., in an interrogator system). There is also no need to control the polarization across the optical path (e.g., by using polarization-maintaining fibers) [26,27,28]. An optical fiber under certain conditions may become sensitive to the position of the polarization plane of the input light. As already mentioned, the most common cause of polarization sensitivity is the disruption of its cylindrical symmetry. It should be noted here that TFBG structures are formed by tilting the axis of refractive index changes with respect to the normal axis of the optical fiber. It is precisely the disruption of such cylindrical symmetry. After inscribing such a structure, the optical fiber loses cylindrical symmetry and becomes sensitive to the polarization of the input light. The light entering such a structure is coupled both to a back-propagating core mode and to several modes propagating in the cladding in a direction consistent with the propagation direction of the input light.

TFBG structures are already known to have many applications as sensors of physical quantities [29,30] and biosensors [31,32], for example, for cancer diagnosis using epithelial growth factor receptor as the biomarker [33,34]. Most often, such periodic structures can be used as sensors only after appropriate preparation of the surface of the fiber in which they are inscribed. This requires specific substances to be sputtered onto the optical fiber cladding surface, for example, lutetium bisphthalocyanines for nitrogen dioxide measurement [35], a gold layer for bacterial density monitoring [36], graphene oxide for relative humidity measurement [37], polyvinyl alcohol for relative humidity sensing [38], graphene oxide and staphylococcal protein A for human IgG detection [39], magnetic fluid for magnetic field measurements [40], Au nanoparticles for mercury ion detection [41], and indium tin oxide for vector twist measurement [42]. Refractometers using TFBGs are also a separate group [43,44,45,46]. However, all proposed RI change sensors using TFBGs are inherently sensitive to changes in the state of the input light polarization plane. This requires knowledge of the input light polarization and its control. Alternatively, as is done in most cases, tests are performed for a given orientation of the polarization without careful analysis of its position, which is cumbersome and contributes to measurement errors for very precise measurements.

If polarized light is introduced into the optical fiber with an inscribed TFBG structure, the spectrum in the range of cladding modes will change, depending on the rotation angle of the polarization of the light. The effect of the polarization rotation angle on the spectral characteristics of TFBG structures can be used to measure the rotation angle and twist [47]. Measurements of many physical quantities require great care and control of the polarization state of the input light, which improves the sensitivity of measurements such as refractometry [48]. However, if the polarization state is not controlled, it will greatly affect the accuracy of the refractometric measurements. One way to reduce the influence of variations in the polarization angle of the input light during measurements is to use unpolarized light. Another way is to precisely control the rotation angle of the input light polarization, which is difficult to do in practice. In this paper, a method was introduced to minimize the effect of changing the input light polarization angle on the spectral characteristics of sensors based on TFBG structures. The method of minimizing the effect of changes in polarization consists of the fabrication of a new TFBG structure, which is insensitive to the change in light polarization.

In our work, we propose to take advantage of the modification of the uniform FBG structure by slightly tilting its diffraction planes as well as performing a twist within the structure itself. In this way, so-called twisted tilted fiber Bragg gratings (TTFBG) are obtained [49]. By twisting the structure of the whole TTFBG, a significant reduction in the sensitivity to the input light polarization angle is obtained. Additionally, this effect can be enhanced by using two TFBG structures rotated with respect to each other by a certain angle. For this kind of combination, we propose the perpendicular tilted fiber Bragg grating named P-TFBG. This is of great importance in practical applications where changes in the plane of polarization of light entering the structure can vary. This affects the optical spectrum of light propagating through such a structure. In this paper, we report the results of using these new T-TFBG and P-TFBG structures to measure the refractive index.

## 2. Materials, Instruments and Methods

### 2.1. Principle of FBG and TFBG Sensing Mechanism

**Figure 1:**
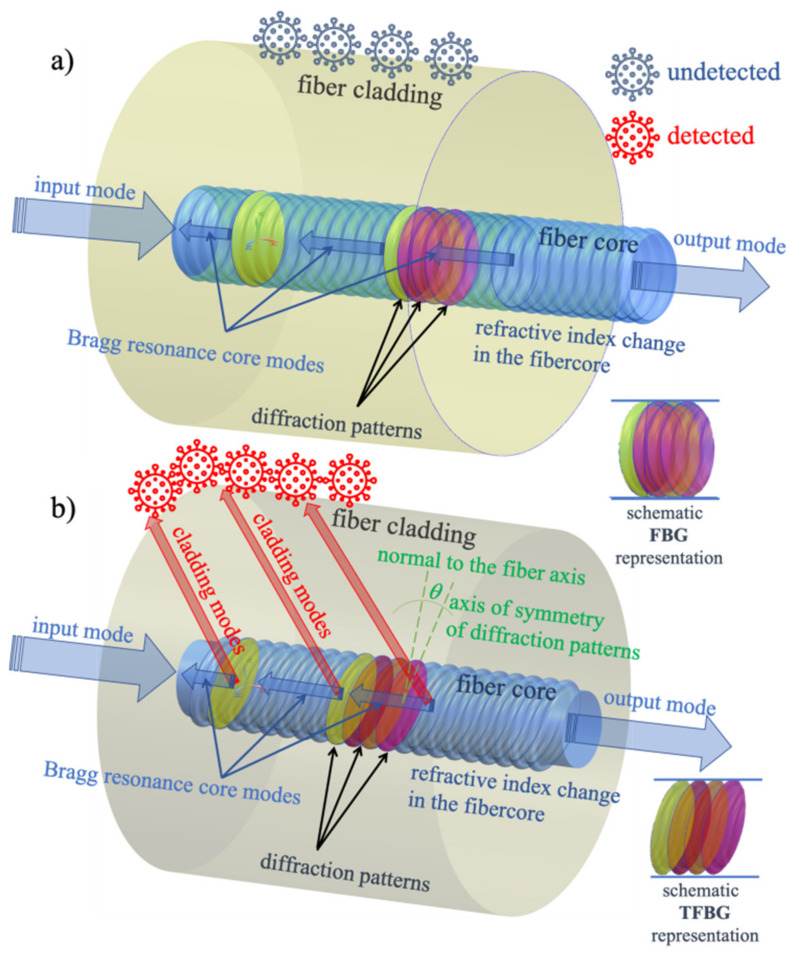
Comparison of the possibility of using FBG and TFBG in the detection of refractive index changes: (**a**) FBG, (**b**) TFBG.

Mechanism of using FBG as a sensor for the refractive index changes caused by (e.g., pathogens) is shown in Figure 1a. When FBG is immersed in analyte, a response in the form of a Bragg wavelength shift occurs, which is caused by a change in the refractive index [50].

Conventional FBG has very low sensitivity to changes in the surrounding medium RI because the grating structure is inscribed in the core of the fiber and the cladding provides an additional obstacle/barrier to the interaction of light propagating through the FBG perturbated core with the medium surrounding the outside border of the fiber. Therefore, etching of a portion of the fiber is often used to allow the analyte the best access to the fiber core [51,52]. Additionally, hydrofluoric acid (HF) is used to etch the fiber cladding at the position of the Bragg grating, which intensifies the evanescent field interacting with the ambient surroundings of the fiber [53]. In some applications of FBGs (e.g., as biosensors), a certain part of the core must also be etched [54].

In our work, we propose that the diffraction planes of the periodic Bragg structure are tilted gently at a certain angle. In this way, we cause some light to start propagating through the cladding and at the cladding-surrounding medium boundary itself (Figure 1b). In this way, no etching of the cladding is needed because the cladding itself is the light carrier and its contact with the surrounding medium causes a conversion of the spectral signal of the optical periodic structure.

Therefore, in our work, we propose an additional procedure in the form of twisting the diffraction planes of the TFBG. Such a structure was first proposed in [49]. On the other hand, we also proposed the creation of a P-TFBG that is based on inscribing two TFBGs twisted with respect to each other by a certain angle. These two solutions make it possible to perform accurate RIU measurements independently from the input light polarization angle. To our knowledge, this is the first work using this type of structure for precise measurements of refractive index changes.

### 2.2. Sensor Fabrication Method

In this paper, an attempt was made to fabricate a TFBG structure that will be insensitive or have minimized sensitivity to changes in light polarization. However, as mentioned earlier, simply twisting the structure is not an optimal solution. The change in the amplitude of the transmission dips, related to the cladding-coupled modes, is smaller, and the twist angle of the TFBG structure is larger [49]. To achieve a reduction in polarization sensitivity, it is necessary to induce a twist on an existing TFBG element. This, in turn, requires the use of systems for setting and precise control of the twist angle of the optical fiber. To this end, a method has been used to produce structures that will already be twisted by themselves by a specific angle.

**Figure 2:**
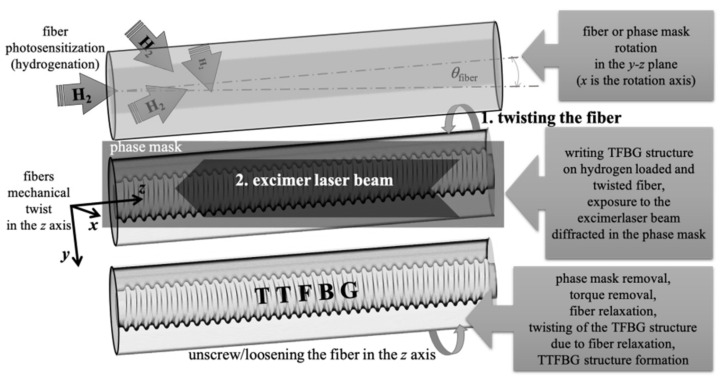
Scheme showing how the TTFBG structure is produced.

Figure 2 shows the method of manufacturing such structures. The method was then applied to produce actual TFBG structures, which, additionally, due to their twist, were marked with the symbol TTFBG.

**Figure 3:**
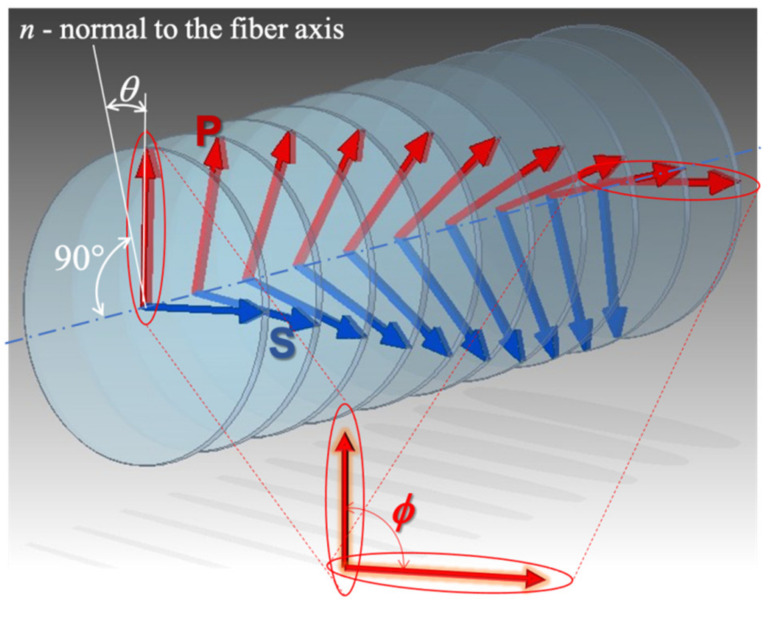
Twisting of the TFBG structure by the angle *ϕ*. Red arrows indicate P-type polarization, blue arrows correspond to S-type polarization.

In addition to tilting the refractive index, modulations will be twisted along its length by a certain angle *ϕ*, according to Figure 3.

Standard single-mode fibers on which TTFBG structures will be inscribed or doped in the core with germanium dioxide GeO_2_ at a level of 3%. It is sufficient to obtain refractive index changes only at a level of 10^−5^. To increase the level of refractive index changes in the core, the level of doping must be increased several times, which is technologically complicated. All structures described in this paper were fabricated by photosensitizing optical fibers in a hydrogen atmosphere. The Bragg gratings used were inscribed on fibers hydrogenated for a period of seven days at a pressure of 200 bar and a temperature of 23 °C. This process allowed for the diffusion of hydrogen atoms into the core of the optical fiber, thus sensitizing it to light in the UV range. As a result, refractive index changes of 10^−2^ were achieved.

All the described structures with tilted refractive index modulations have one feature in common, which imposes certain limitations in detection systems. Thus, if polarized light is introduced into the fiber with TFBG, the transmission corresponding to the selected cladding modes will change, depending on the polarization state of this light. The influence of the input light polarization on the spectrum of TFBG structures thus determines the need to control the state (e.g., the angle) of the polarization plane. Therefore, the research leading to a reduction or elimination in the influence of the input light polarization angle on the optical parameters (e.g., the shape of transmission characteristics) of tilted periodic structures is justified.

It should also be noted that on a mechanically twisted fiber, the TFBG structure is actually formed. The next stage is therefore the release, the so-called relaxation of the fiber. As twisting takes place within the elastic limits of the fiber, the release of the twisting moment causes the fiber to return to its untwisted state. In turn, this causes the TFBG structure that was written on the twisted fiber to transform into a TTFBG structure as a result of fiber relaxation.

**Figure 4:**
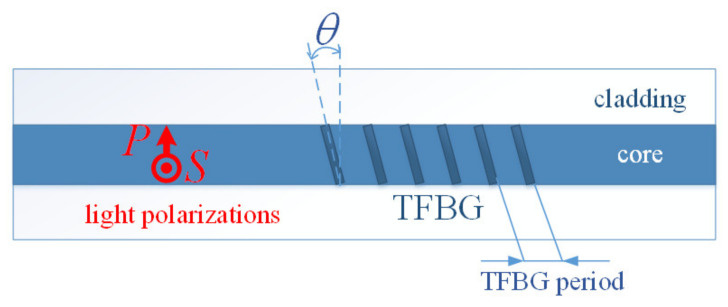
Marking of the two orthogonal polarization states in the TFBG structure.

In the TFBG structure, the property of splitting the mode into two separate transmission dips, understood as extremes in the spectral characteristics, for two polarizations is possessed by asymmetric modes LP*_1m_*. The mode splitting is the effect of inclination of the plane of refractive index change in the optical fiber core. The polarized light introduced into the optical fiber, depending on its orientation in relation to the tilted grating planes, causes changes in the amplitudes of the divided mode spectral characteristics. This effect is called dual-peak transmission. This property can be used for rotation, twist, and bending measurements [47,55,56]. The two resulting orthogonal polarization states are referred to as P and S, with the P-polarized state defining linearly polarized light propagating in the plane formed by the *y*–*z* axes (i.e., in the plane in which the reflecting surfaces are inclined, i.e., tilt planes). On the other hand, the S-polarized state defines linearly polarized light, which propagates perpendicularly to this plane [57,58]. The spatial orientation of both states is shown in Figure 4.

The difference between the wavelengths of the S and P resonances is larger for higher-order modes. At the same time, this difference is larger for gratings with larger angles of the reflecting planes [59]. Increasing the distance between adjacent symmetric LP_0m_ and asymmetric LP_1m_ modes is possible by decreasing the diameter of the optical fiber core on which the TFBG is inscribed [60]. TFBGs written on multimode fibers, the so-called multimode fiber tilted Bragg gratings, have larger core diameters, which results in completely different spectral changes under polarization state changes [61]. A detailed analysis of the properties of the structure under polarization change in the context of the change in spectral parameters corresponding to the cladding modes is presented in [24].

**Figure 5:**
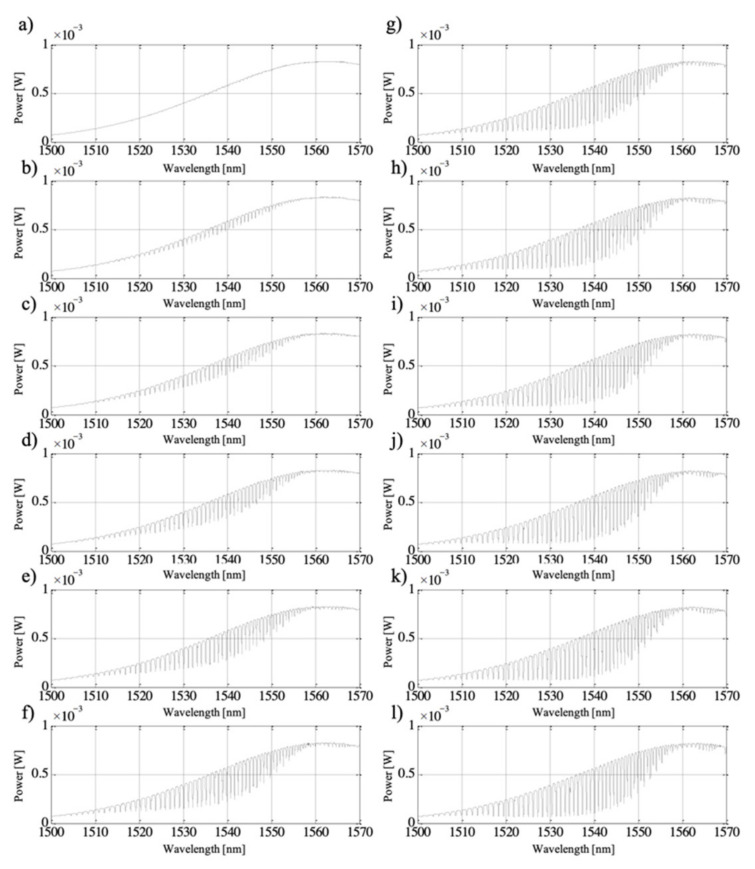
The process of TFBG structure formation on an optical fiber twisted by an angle equal to 180°. The figure shows the transmission characteristics measured at different times after switching on the laser: (**a**) 4 s, (**b**) 8 s, (**c**) 12 s, (**d**) 16 s, (**e**) 20 s, (**f**) 24 s, (**g**) 28 s, (**h**) 32 s, (**i**) 36 s, (**j**) 40 s, (**k**) 44 s, (**l**) 48 s.

For the aim of this work, the TFBG was fabricated with a period *Λ* = 525 nm and *θ* = 5° to exhibit coupling up to 30 modes, as depicted in Figure 5. This figure shows the successive stages of the process of minima formation on the transmission characteristics of the optical fiber twisted by 180° during TFBG manufacturing. The structure, the characteristics of which are presented in Figure 2 and Figure 3, was inscribed with a Coherent Inc. laser operating at a power of 100 mJ and a repetition rate of 100 Hz. An increase in the fiber exposure time caused an increase in the individual minimum on the transmission spectral characteristics, which is caused by an increasingly stronger coupling of light to the cladding modes. The manufacturing time of the whole structure on the twisted fiber was 48 s.

## 3. Results and Discussion

### 3.1. Polarization Insensibilization Results

**Figure 6:**
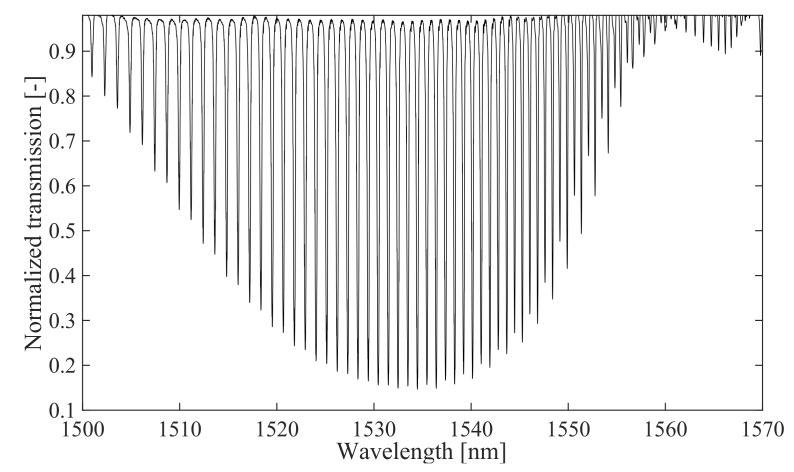
Spectral characteristics of the TFBG structure: *θ* = 5°, *ϕ* = 0°.

First, to demonstrate the effect of the twisting of the structure on the dependence of its transmission spectrum on the polarization of the input light in the first step, we performed a study in which the TFBG was twisted with a known angle *ϕ*. A TFBG structure with a tilted angle *θ* = 5 was fabricated for the study. The transmission characteristics of the whole structure without induced twist are shown in Figure 6.

**Figure 7:**
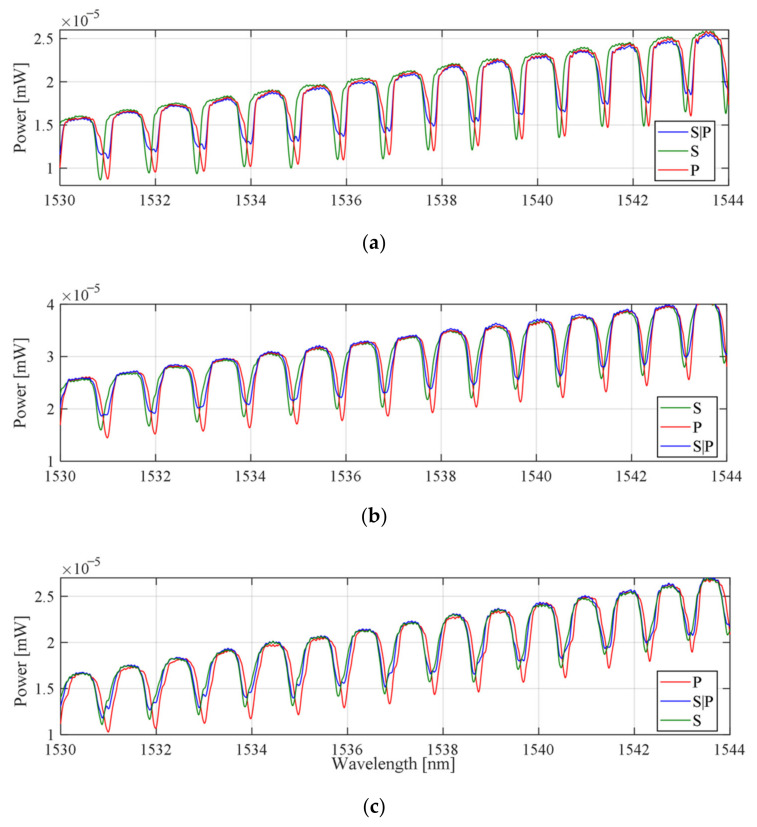
Results of spectral measurements of TFBG *θ* = 5° for three states of polarization of the input light for wavelengths: (**a**) *ϕ* = 0° untwisted, (**b**) *ϕ* = 45°, (**c**) *ϕ* = 90°.

The spectral evolution due to changes in the polarization angle for the untwisted structure (*ϕ* = 0°) and twisted with angle *ϕ* = 45° and *ϕ* = 90° in the spectral range 1530–1544 nm are summarized in Figure 7. The three spectral characteristics were measured for three different values of the input light polarization angle. P-type polarization rotated by an angle of 45° (which corresponded to S|P-type, and polarization rotated by 90°, which corresponded to S-type).

Since in our work we proposed the use of a structure composed of modified TFBG structures for the detection of subtle changes in refractive index, it is important to eliminate the main factors causing additional changes in the spectral characteristics of such Bragg structures.

**Figure 8:**
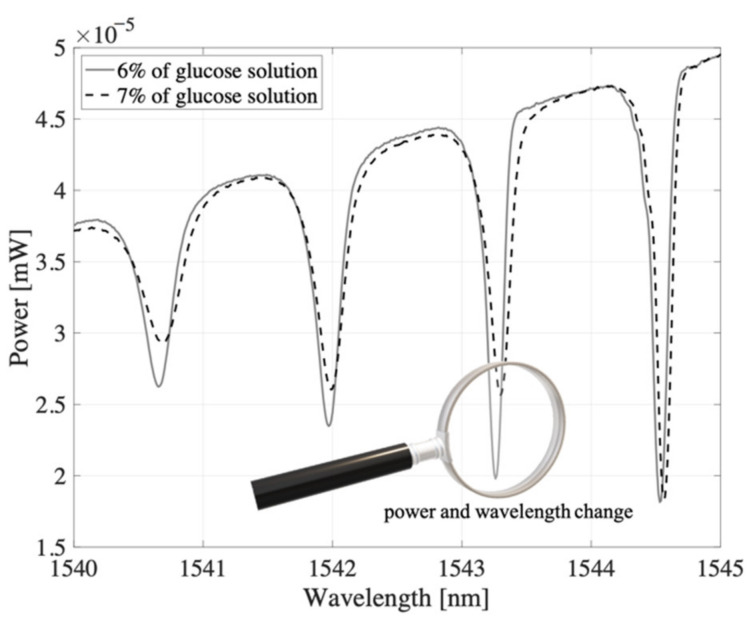
Evolution of spectral characteristics measured in transmission mode induced by a 1% change in glucose concentration (preliminary tests). TFBG: *θ* = 8°.

To estimate the sensitivity of TFBG (not TTFBG and not P-TFBG) to changes in refractive index values, we created a structure with an angle *θ* = 7 and then investigated how selected dips that are part of the TFBG spectrum responded to changes in the RI of the medium. To do this, we produced an aqueous glucose solution, and by controlling the mass of the whole solution and the mass of the glucose, we determined the exact refractive index value. Figure 8 shows the spectral range corresponding to the wavelengths of the selected TFBG peak that, with a tilt angle *θ* = 7, responded most strongly to changes in RIU.

A 1% change in glucose concentration induced a change in refractive index of 0.0015 RIU. In this first test, we did not define sensitivity as the ratio of change in optical power to change in RIU because the value of the signal power depends on how strongly we excite the fiber (i.e., what the input power is).

This will be different for a laser diode (@1550 nm) and different tunable laser (approximately @1550 nm). Therefore, for the rest of the paper, we switched to a change in transmission instead of a change in power. In our preliminary measurements, a change in RIU on the order of 10^−6^ resulted in a change in the transmission coefficient of 88.88·10^−6^, which corresponds to an average sensitivity across the range of 88.88/RIU. For example, if one were to introduce a signal from a light source into such a sensor, which for this wavelength gives a power equal to 100 W, then the power difference with the given RIU change would be 8.88 nW/10^−6^ RIU. Changing the concentration of glucose from 6% to 7% resulted in a change in RI from 1.3418 to 1.3433, obtaining a difference in RIU of 0.0015. For example, changing the power of the selected peak from 19.8 nW to 26 nW when the maximum value was 45 nm (the change in t in this case is from 0 to 1) will result in a change in *t* from a value of 0.44 to a value of 0.58, showing a difference of Δ*t* = 0.133.

**Figure 9:**
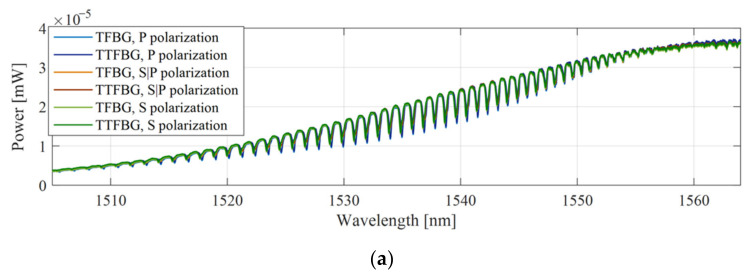

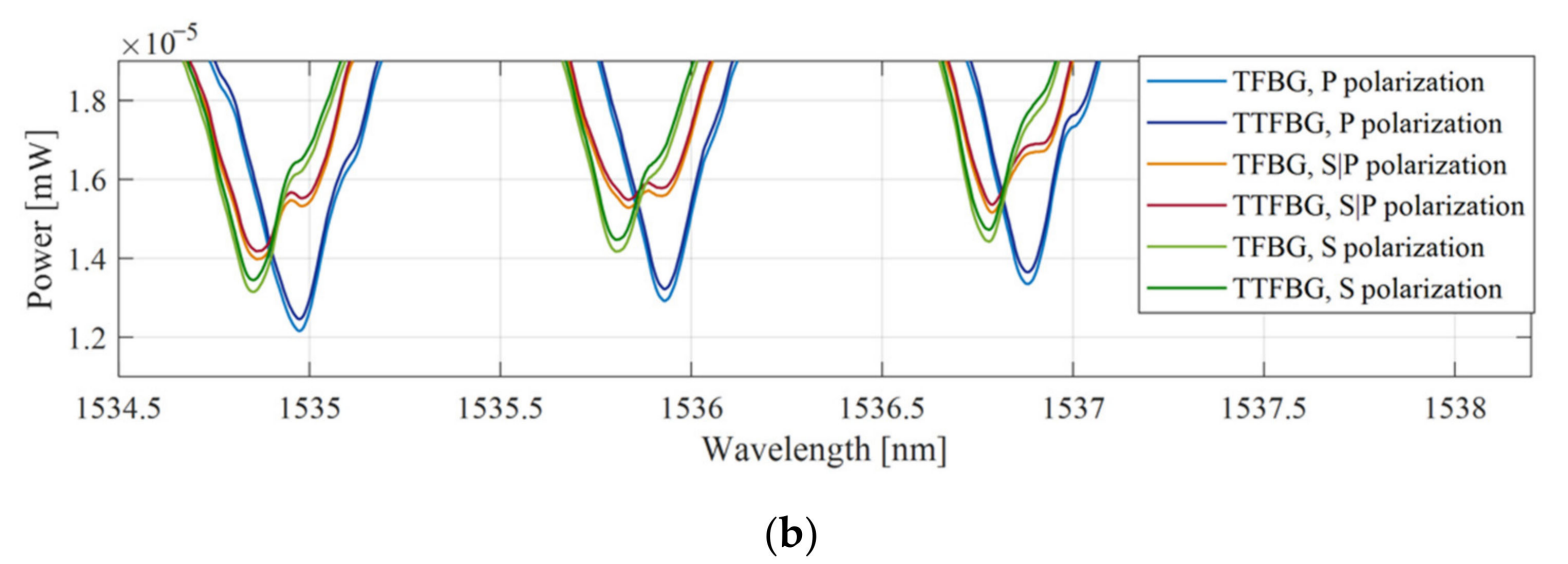
Comparison of the spectral characteristics of two structures TTFBG with the twist angle equal to 90° and TFBG twisted for the time of measurements by the angle equal to 90°: (**a**) the whole spectral range, (**b**) selected spectral range corresponding to three selected cladding modes.

The first study of the effect of the rotation angle of input light polarization on the spectral characteristics was performed for TTFBG structures with a rotation angle of 90°. Measurements were performed for three input light polarization states: P, S|P, and S. For comparison, analogous measurements were also performed for the TFBG structure twisted by the same angle of 90°. The measurement results are presented in Figure 9.

The changes in the TFBG and TTFBG spectra have the same characteristics. The effect of minimizing the sensitivity to a change in the angle of rotation of the input light polarization can be obtained by twisting the TFBG structure for the duration of the measurements.

The same effect can also be obtained by using a TTFBG structure with the same twist angle as the TFBG. It should also be noted here that, in contrast to the publications on the use of TFBGs, in TTFBG-based twist sensors, only the twist of the tilted structure itself is analyzed and not, for example, the twist of the polarization-maintaining optical fiber and the grating. Although twisted structures are known for long-period gratings [62], the TTFBGs described in this section are one of the first presentations of twisted tilted fiber Bragg gratings.

**Figure 10:**
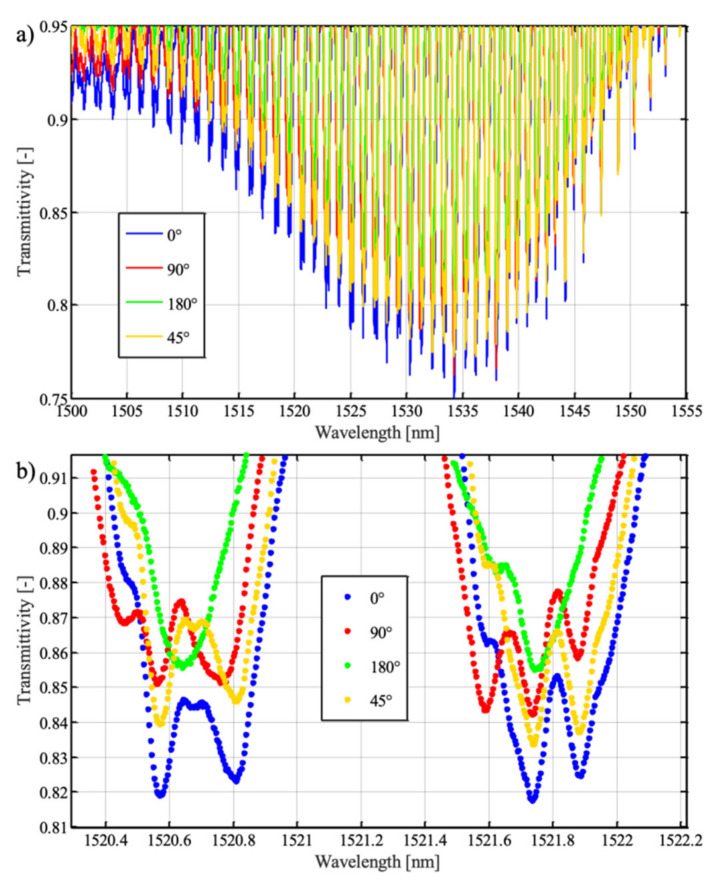
Comparison of the spectral characteristics of four TTFBG structures differing in the *ϕ* angle: (**a**) the entire spectral range, (**b**) a selected spectral range corresponding to two selected cladding modes.

The fabricated TTFBGs were then subjected to spectral studies under different polarization conditions. To determine the degree of twist of the structure, appropriate angular markers were introduced between which the twist angle *ϕ* was measured. The following structures were investigated: TFBG (TTFBG *ϕ* = 0°), TTFBG *ϕ* = 45°, TTFBG *ϕ* = 90°, and TTFBG *ϕ* = 180°. All inscribed gratings had a refractive index modulation angle of *θ* = 5°. The results of measurements for two spectral ranges, full (a) and narrowed for selected cladding modes (b), are summarized in Figure 10. The direct effect of twisting the structure is to reduce the variation in the transmission coefficient for individual cladding modes with changes in input light polarization.

**Figure 11:**
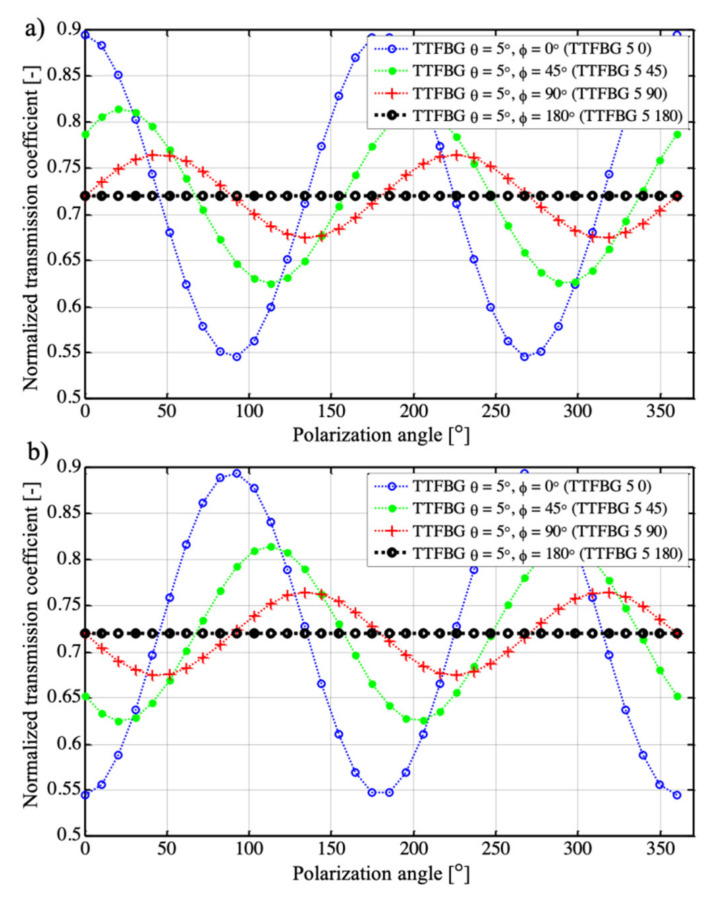
Results of numerical calculations of changes in the transmission coefficient of the TTFBG structure for various ϕ angles: (**a**) for P-type mode, (**b**) for S-type mode.

**Figure 12:**
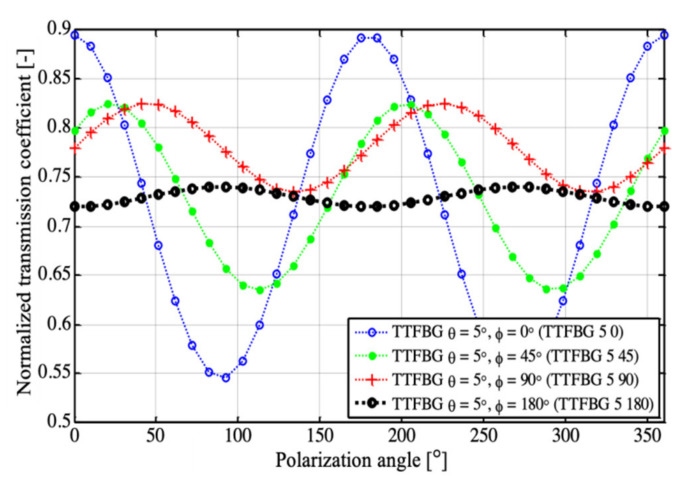
Measurement results of the transmission coefficient variation of the TTFBG structure for different twist angles ϕ.

The graphs shown in Figure 11 refer to the variation in the transmission coefficient value of the selected cladding mode. The graphs were obtained by numerical calculation by dividing the structure into 50 elements. Each such element was assigned an appropriate transmission spectrum. Figure 12 shows the changes in P-mode and S-mode transmission due to changes in the polarization state of the introduced light for a TFBG structure. A TTFBG structure twisted along its length by 45°, 90°, and 180°. For comparison, all structures had the same length *L* = 10 mm.

To illustrate the dependence of the spectrum on the polarization twist, the diagrams in Figure 11 show the transmission coefficients for the minimum coming from a selected mode. The most important stage of numerical calculations is to determine the transmission coefficient of a uniform, non-tilted grating *T_ϕ_*(*α*) for each polarization state represented by the angle *α* of linearly polarized light. The power transmission coefficient is defined as the ratio between the output and input powers. Such transmission was determined using measurements made for a conventional untwisted TFBG structure of 1 cm in length and an *θ* angle equal to 5°. The transmission of a twisted grating was determined as the product of the transmission of 50 homogeneous TFBG structures of 1/50 cm in length. In this case, the elementary transmission coefficient of a homogeneous TFBG structure will be defined by Equation (1):

(1) $$T_{\phi }\left(\alpha \right)=\sqrt[{50}]{T\left(\alpha \right)}$$

Accordingly, the transmission coefficient of a grating twisted by an angle equal to 90° will be determined by Equation (2):

(2) $$T_{0-90}\left(\alpha \right)=T_{\phi }\left(\alpha \right)\cdot T_{\phi }\left(\alpha +\frac{1}{50}90{}^{\circ}\right)\cdot T_{\phi }\left(\alpha +\frac{2}{50}90{}^{\circ}\right)\cdot \ldots \cdot T_{\phi }\left(\alpha +\frac{49}{50}90{}^{\circ}\right),$$

whereas in the case of a structure twisted by an angle equal to 180°, its transmission coefficient was determined using Equation (3):

(3) $$T_{0-180}\left(\alpha \right)=T_{\phi }\left(\alpha \right)\cdot T_{\phi }\left(\alpha +\frac{1}{50}180{}^{\circ}\right)\cdot T_{\phi }\left(\alpha +\frac{2}{50}180{}^{\circ}\right)\cdot \ldots \cdot T_{\phi }\left(\alpha +\frac{49}{50}180{}^{\circ}\right).$$

When the grating was twisted by an angle *ϕ* = 180°, the individual elementary gratings were twisted by angles 0°, 180°/50, 2 180°/50, 3 180°/50, 4 180°/50…, 49 180°/50. Figure 12 shows the results of numerical calculations of the transmission coefficient performed for the minimum obtained from the selected cladding mode.

For the untwisted structure, the shape of the modal transmission variations was asymmetric. Therefore, both the direction of polarization rotation and the direction of twist of such a grating cannot be determined from its spectral characteristics. In the case of a structure for which *ϕ* = 45° when the rotation angle of the polarization plane changes in the range from −22.5° to +22.5°, such discrimination is possible. The twist angle of such a structure can also be determined. A grating twisted by 180 degrees along its length does not show any sensitivity to polarization.

The effect of changes in the angle of rotation of the input light polarization on the transmission spectra in the range from 0° to 90° was significantly reduced when the twist angle of the TTFBG structure increased. To determine the effect of polarization on the sensor response, a parameter was determined in the form of the transmission sensitivity coefficient to a change in the polarization plane rotation angle in the range from 0° to 90°, which was defined by Equation (4) in the following form:

(4) $$K_{\alpha }^{t}=\frac{\Delta t}{\Delta \alpha_{\left|0-90\right|}}$$

where $\Delta \alpha_{\left|0-90\right|}$ specifies the change in the polarization plane angle in the range from 0° to 90°. Additional information about the decrease in sensitivity to the angle of rotation of the input light polarization plane is provided by a parameter taking into account the reference value of the transmission coefficient, in this case, the initial value. Therefore, the parameter determining the relative percentage change of the transmission coefficient on the polarization angle was defined and determined by the following relation, which specifies the change in the polarization plane angle in the range from 0° to 90°:

(5) $$\delta T_{\phi }=\left(\frac{T_{\phi \max}-T_{\phi \min}}{T_{|0}}\right)$$

where *T*_max_ and *T*_min_ denote the minimum and maximum values of the transmission coefficient of the TTFBG structure in the examined range of changes of polarization of the input light, respectively, and *T*_|0_ is the value of the transmission coefficient of the structure for the reference state, for which *α* = 0. The measurement results corresponding to the cases modeled in Figure 11 are shown in Figure 12.

The values of parameters determining the sensitivity of new TTFBG structures to polarization are summarized in Table 1. In the case of existing TFBG gratings, changing the angle of the plane of polarization from 0° to 90° significantly affects the light propagating through the structure, resulting in changes in the amplitude of the peaks originating from individual cladding modes.

**Figure 13:**
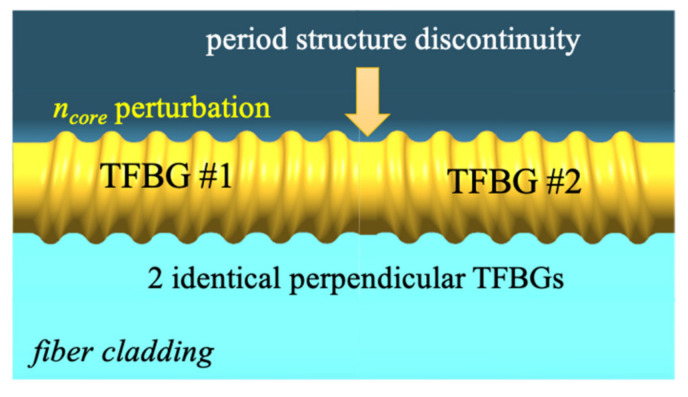
Structure composed of two identical TFBG twisted in relation to each other.

This results in a change in the transmission coefficient of the structure. For a TFBG grating (i.e., one in which there is no twist (*ϕ* = 0°)), the parameter defining the relative change in the transmission coefficient value is at the level of *δT_ϕ_* = 39% (with a range of transmission coefficient changes from 0.546 to 0.895). This corresponds to a structure polarization sensitivity factor of 3.9 × 10^−3^ 1/°. The TTFBG structure whose twist angle is 45° is already clearly less sensitive to polarization than TFBG. The relative value of the change in the transmission coefficient of TTFBG *θ* = 5°, *ϕ* = 45° is already only 23.76%, which results in a decrease in the polarization sensitivity factor to a value of 2.1 × 10^−3^ 1/°. An increase in the twist angle of the TTFBG *ϕ* causes a further decrease in the value of the transmission coefficient with changing polarization of the input light. For TTFBG *θ* = 5°, *ϕ* = 45° the parameter *δT_ϕ_* = 2.78%, determining that the percentage change of the transmission coefficient is more than 14 times smaller than for TFBG structures. Additionally, the transmission sensitivity coefficient to a change in the polarization plane rotation angle for such a structure undergoes a significant (17-fold in comparison with TFBG structures) decrease to the value of K_α_^T^ = 0.2 × 10^−3^ 1/°. However, it should be noted that as the angle increases, the minima of the spectral characteristics of the TTFBG, corresponding to individual cladding modes, also decrease. This should be taken into account in measuring systems using this type of structure as transducers of RI changes caused, for example, by the presence of pathogens. For this reason, in the following section, we proposed simplifying such a system into two TFBGs rotated with respect to each other by an angle of 90°. This approach eliminates the significant decrease in the height of an individual minimum on the transmission spectral characteristics. We named this arrangement P-TFBG (perpendicular TFBG) and present its idea in Figure 13.

**Figure 14:**
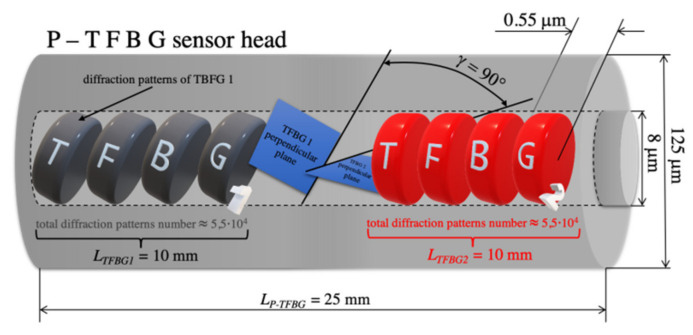
Idea of determining the *γ* angle in the P-TFBG structure.

**Figure 15:**
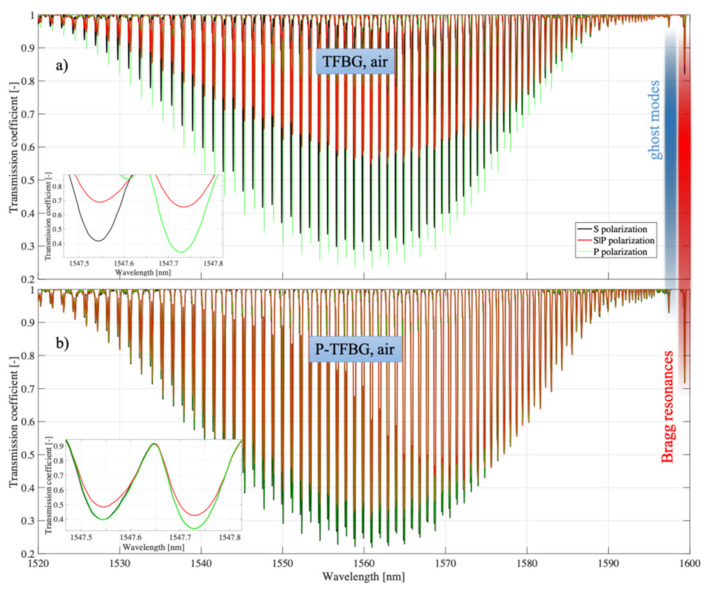
Spectral characteristics measured in air for three input light polarization states: (**a**) TFBG, (**b**) P-TFBG system.

The angle by which the two TFBG structures were twisted with respect to each other was 90°, but to show precisely how this angle was defined, it is marked in Figure 15 as *γ*. To define it, we determined planes perpendicular to the diffraction patterns of the TFBG. Since these are planes with the same spatial orientation with respect to each TFBG, the angle between these planes will also be *γ*. We present the idea of determining this angle in Figure 14.

**Figure 16:**
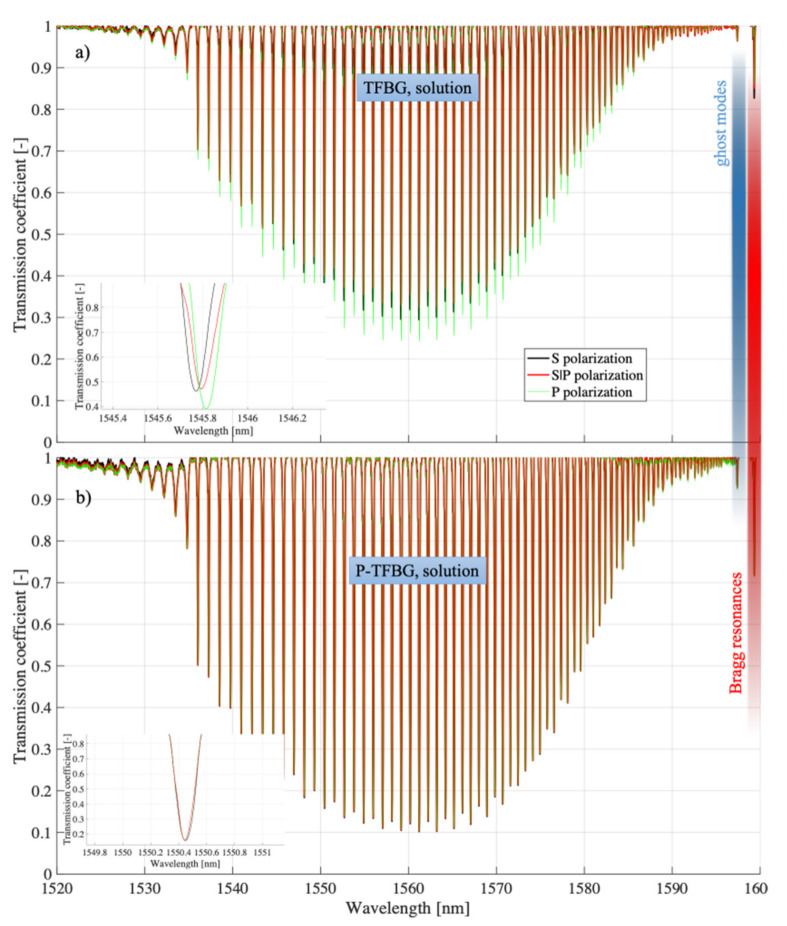
Spectral characteristics measured in solution for three polarization states of the input light of (**a**) TFBG, (**b**) P-TFBG system.

Figure 15 summarizes the spectral characteristics of the TFBG and P-TFBG structures, all having angles *θ* = 5°, measured in air, without contact with the aqueous solution. We performed the measurements for the three polarization states S, P, and S|P, as labeled in Section 3. A strong change in the response of the spectrum to a change in polarization was evident. To illustrate this effect, we present a selected section of the spectrum in the inset of Figure 16.

For comparison, Figure 16 collects together analogous characteristics, but already obtained from measurements in which TFBG and P-TFBG structures were immersed in glucose aqueous solution, whose refractive index was 1.3471.

**Figure 17:**
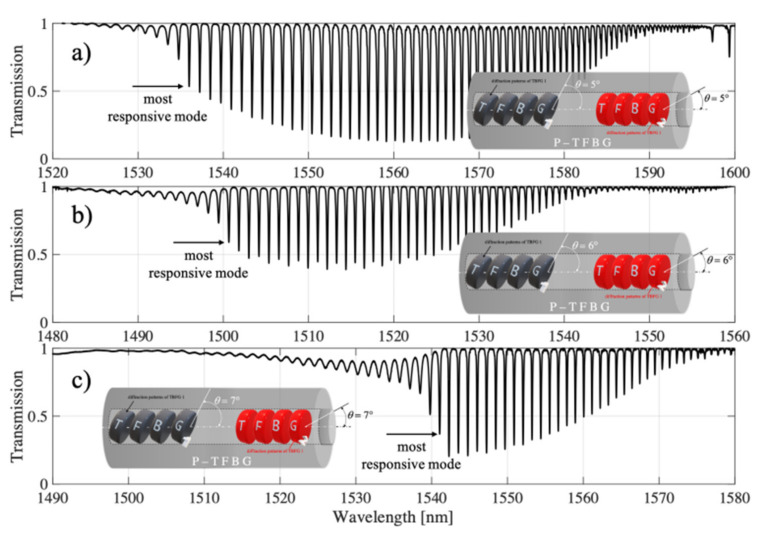
Spectral characteristics of the P-TFBG system for variable inclination angles of TFBG structures of: (**a**) 5°, (**b**) 6°, (**c**) 7°.

All the structures in Figure 17 had angles *q* = 5°. In summary, it can be concluded that the presented experimental results demonstrate the possibility of reducing the polarization sensitivity of TTFBGs compared to the TFBG structures currently used, especially when measuring refractive index changes. This section presents the structures of tilted Bragg gratings, which are formed by twisting the optical fiber during the recording process with UV light. The twist present in the TTFBG structure results in a reduction in its sensitivity to the polarization of the input light. Theoretically, in the case of a conventional, symmetrical TTFBG structure twisted by an angle equal to 180° it becomes completely insensitive to the influence of polarization. In practice, however, a slight sensitivity to light polarization also occurs in TTFBG structures, as demonstrated by the analysis of the experimental results presented in this chapter. The reason for this phenomenon is the nonuniform shape of the excimer laser power distribution along the length of the optical fiber section on which the entire TTFBG structure is written. It was also shown that an array of two parallel P-TFBG structures had a reduced polarization sensitivity. To be able to compare TTFBG and P-TFBG structures, we produced them on the basis of the same TFBG structures (i.e., having the same length *L* = 12 mm and angle of inclination of diffraction planes *q* = 5°). The advantages of P-TFBG over TTFBG were even more apparent when immersed in solution, which is particularly evident when comparing Figure 15 and Figure 16. In solution, the sensitivity of P-TFBG to the polarization of the input light decreases even more. In the remainder of this paper, we have shown that the P-TFBG system can still be optimized, as the *q* angle affects the sensitivity of individual peaks on the transmission characteristic to polarization. We have intentionally included these results later in the paper after analyzing the coefficient changes in the third telecommunication window because the structures produced will be adapted to the results reported in this chapter.

### 3.2. Mode Preselection

In this section, we prove that the proper selection of the mode and the associated minimum on the transmission spectral characteristics is crucial for P-TFBG structures. During the analysis of the different spectral ranges, we noticed that it is possible to improve the sensitivity of the power variations coupled to the individual modes of the P-TFBG structure by changing the angle of the diffraction planes of the two TFBG gratings that make up the P-TFBG array. In Figure 17, we present the comb-like resonances of the P-TFBG system for different tilt angles of the TFBG planes, with the indication of those modes that react most strongly to a change in the refractive index in the range from 1.3461 to 1.3471.

**Figure 18:**
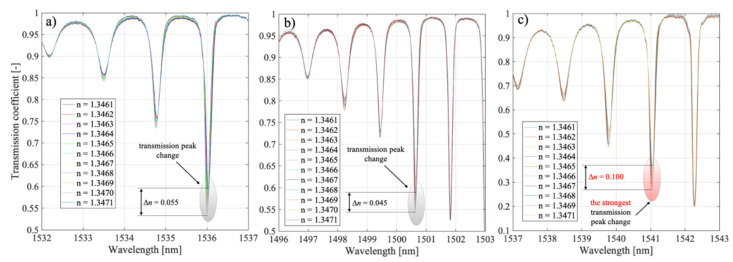
Spectral characteristics of the P-TFBG system in the selected spectral range for variable tilting angles of TFBG structures amounting to: (**a**) 5°, (**b**) 6°, (**c**) 7°.

The preliminary analysis of the spectra of the structures allows us to conclude that they differ significantly in their response to the refractive index sweep. In Figure 18, we have indicated that the strongest reaction modes were @1536 nm for P-TFPB 5°, @1500.06 nm for P-TFPB 6°, and @1541 nm for P-TFPB 7°. The strongest dependence of the transmission coefficient for the central wavelength of the cladding mode is characterized by the structure with P-TFBG 7°.

As seen above, the greatest change in the value of the transmission coefficient was obtained for a grating *θ* = 7°. Additionally, it can be observed that the cladding modes that responded with the strongest changes in the transmission coefficient had a greater amplitude in the case of the TFBG 7° structure than in the cases of TFBG 5° and TFBG 6°.

**Figure 19:**
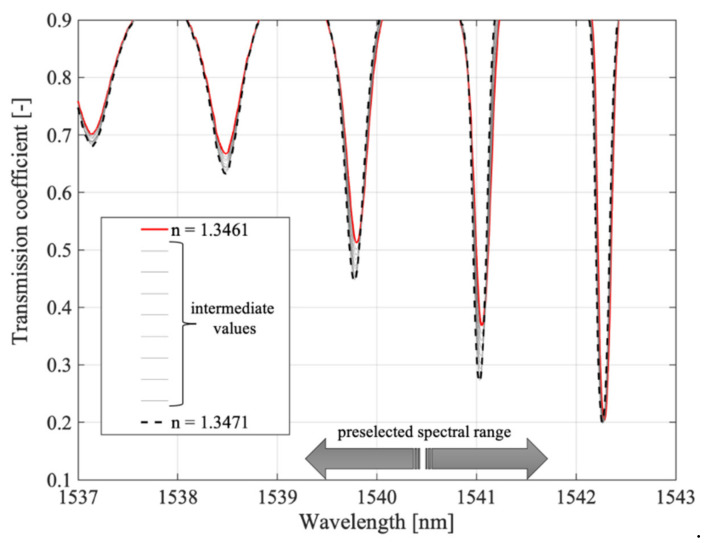
Results of the spectral measurements for modal preselection P-TFBG 7°. Measurements were made in the range of changes RI = 1.3461–1.3471.

Figure 19 shows the evolution of the spectrum with refractive index changes varying by 0.0001. We have deliberately not shown the specific refractive index values in the legend, as this is not relevant for this figure.

**Figure 20:**
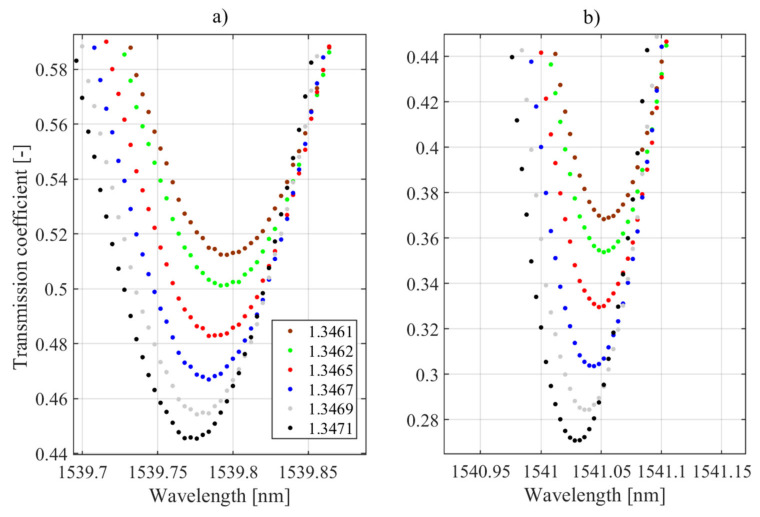
Results of the spectral measurements for TTFBG modal preselection. (**a**) mode @1539.8 nm, (**b**) mode @1541.05 nm.

Indeed, the specific values and the proof of the monotonicity of the changes are presented in Figure 20, which already showed that selected cladding modes had the highest sensitivity to refractive index changes. It is worth noting that the preselection of modes is crucial in the case of P-TFBG.

This is because the individual modes differ significantly in their sensitivity to changes in surrounding RI. Note also that only the value of the mode amplitude changes due to RI changes because the sign of the sensitivity coefficient does not change. This is because the amplitude change of mode @1538.5 nm had the same sign as that of modes @1539.8 and @1541.05 nm.

### 3.3. Refractive Index of Glucose Solution Measurements

It was determined that the estimated value of the refractive index for medical applications for the wavelength intervals in which the cladding modes of the fabricated structures are located is in the range of 1.346–1.347. Using the notation method presented in Figure 2, we fabricated P-TFBG structures presented in Figure 13, characterized by the known position of the cladding modes. Knowing the spectral range of the P-TFBG comb, we created standard solutions using glucose. These solutions had a well-defined refractive index determined by temperature and the percentage of glucose in water. We then placed the P-TFBG structure in these solutions and determined, from the spectra obtained from the measurements, which cladding modes had the highest sensitivity. For this purpose, we determined the transmission sensitivity coefficient of a given mode to a change in refractive index. In this paper, we called this part of the spectral study modal preselection.

In the introduction to this section, we present the effect of changes in the plane of polarization of the input light on the change in the transmission coefficient of TFBG 7 and the analogous P-TFBG 7° system. As we presented in Section 3, when measuring very small changes in refractive index, it is very important to eliminate other factors interfering with the signal as much as possible. Such a factor is undoubtedly polarization, the knowledge and control of which in real measurement systems causes many technical problems. Therefore, it is important to develop a sensor that, as a rule, will be less sensitive to changes in polarization. The spectral characteristics of TFBG and P-TFBG structures (all having angles *θ* = 7°), measured in air and in a glucose aqueous solution, for the selected spectral range described in Section 4 on modal preselection are summarized below. We performed the measurements for the three polarization states S, P, and S|P, as indicated in Section 3. The TFBG and P-TFBG structures were immersed in an aqueous solution of glucose with a refractive index of 1.3471.

**Figure 21:**
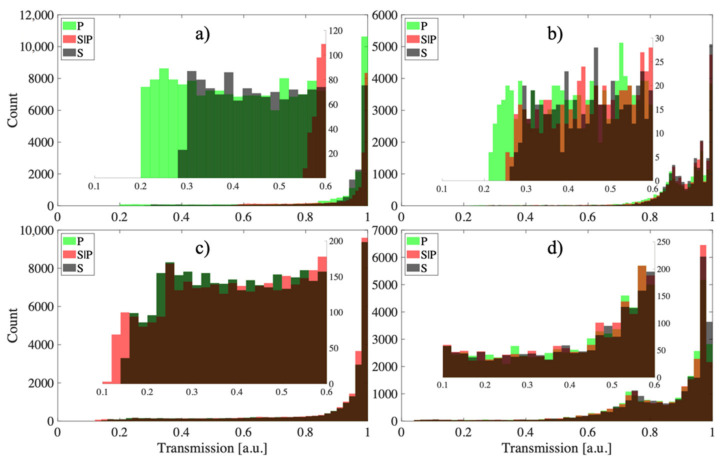
Histograms showing the response of the sensors to changes in P, S|P, and S polarization for the cases: (**a**) TFBG air, (**b**) TFBG solution, (**c**) P-TFBG air, (**d**) P-TFBG.

A strong change in the spectral response to a change in polarization for both structures could be seen. One way to demonstrate the sensitivity of individual structures to polarization is to plot histograms for individual cases of TFBG and P-TFBG placed in air and in glucose solution. The histograms for the three polarization states in Figure 21 show that the TFBG structures had the largest differences in transmission coefficient values. To present the phenomenon of structural parameter change more precisely, Figure 21a–d also have a narrowed region contained in the range from 0.1–0.6 because polarization changes mainly influence the change in the transmission coefficient of structures most strongly in this range.

**Figure 22:**
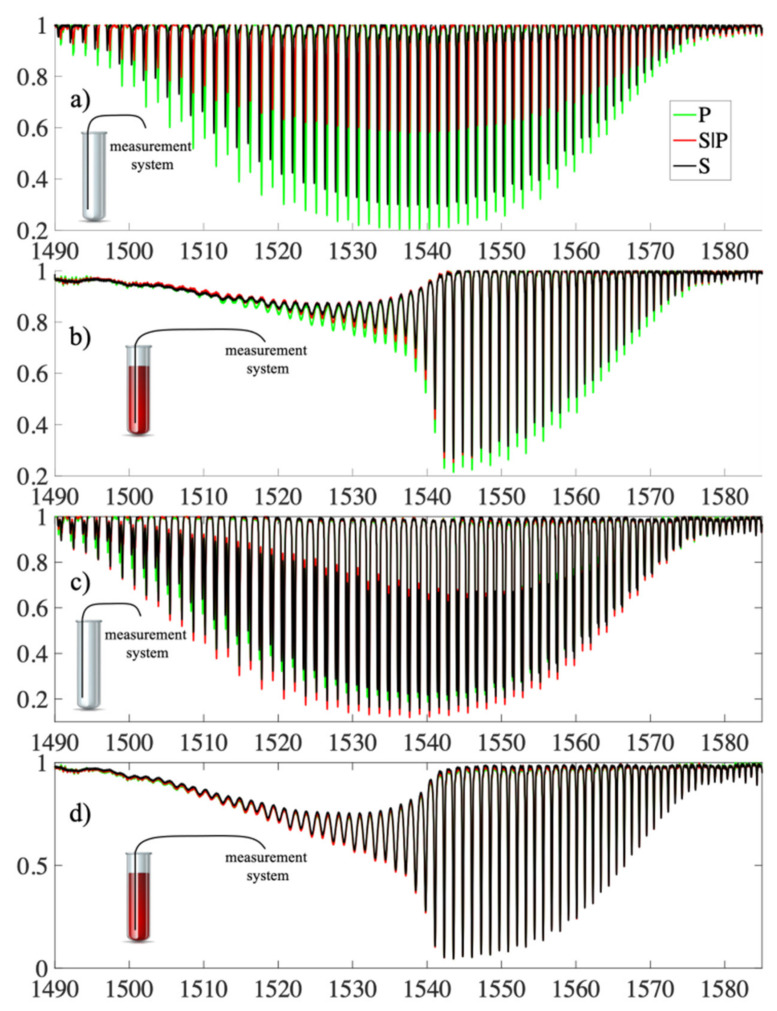
Measured spectral characteristics showing the response of the sensors to changes in polarization P, S|P and S for the cases: (**a**) TFBG air, (**b**) TFBG solution, (**c**) P-TFBG air, (**d**) P-TFBG solution.

**Figure 23:**
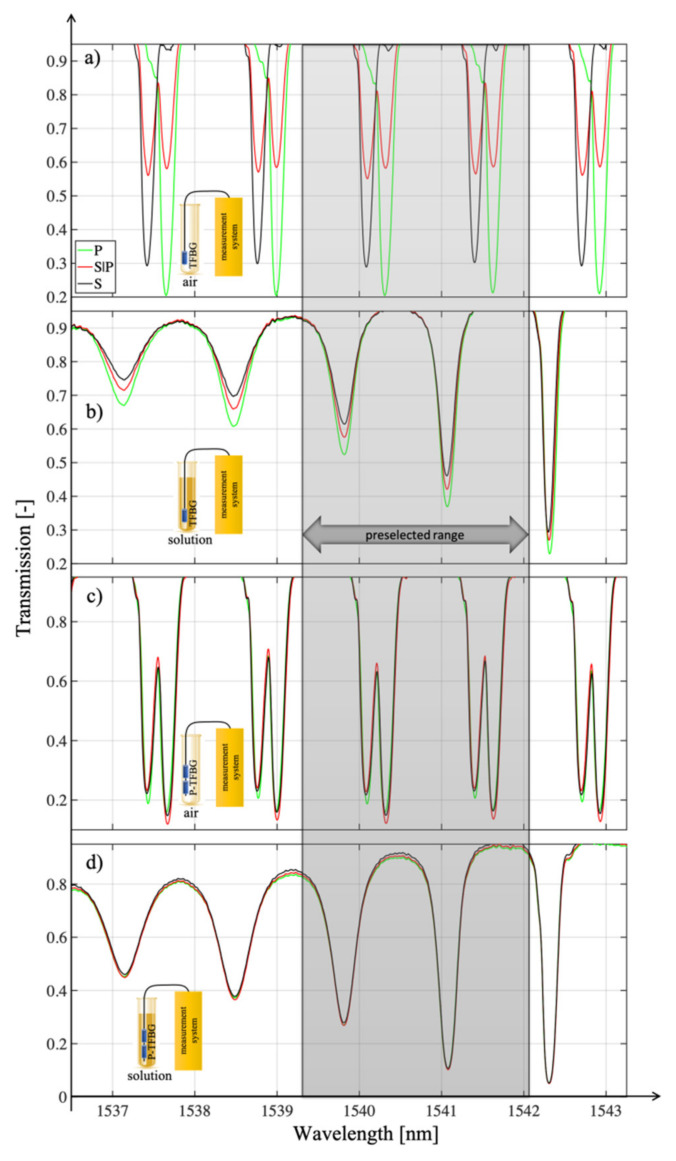
Measured spectral characteristics in the preselected range showing the response of the sensors to changes in P, S|P, and S polarization for the cases: (**a**) TFBG air, (**b**) TFBG solution, (**c**) P-TFBG air, (**d**) P-TFBG solution. All structures had angles *θ* = 7°.

**Figure 24:**
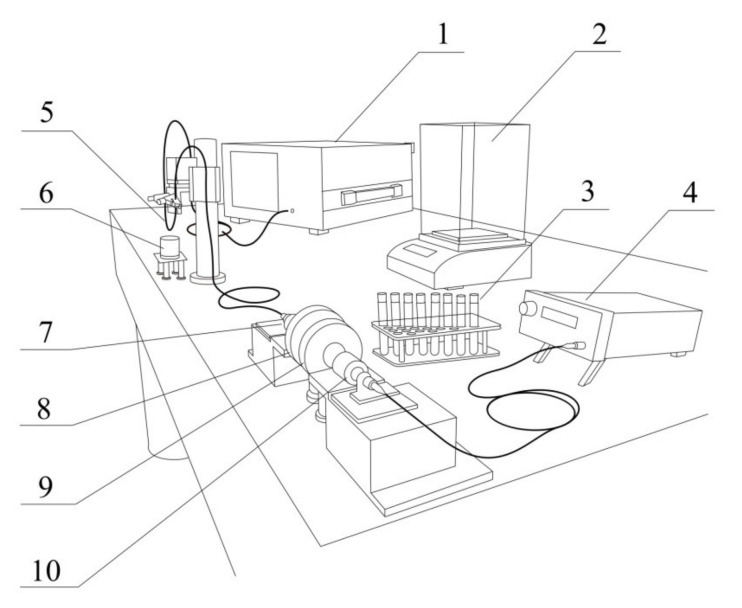
Measurement system scheme: 1—optical spectrum analyzer, 2—electronic scales, 3—test tubes with glucose solutions, 4—superluminescent diode, 5—TTFBG and P-TFBG sensors, 6—test solution, 7—microscope lens, 8—half wave plate mounted in rotation base, 9—polarizer, 10—microscope lens.

The greater the coverage of the histograms in Figure 21, the greater the insensitivity of the sensor to changes in the input light polarization. By analyzing Figure 21, it can be seen that the P-TFBG structures were the least sensitive to changes in polarization. For both polarization changes introduced to the P-TFBG sensor in air and in solution, the changes in its transmission coefficient were significantly smaller than for a TFBG structure with the same input light parameters. The response of the entire spectrum to changes in polarization, already for the selected structures, is shown in Figure 22, which includes the four cases discussed, already selected after the section on modal preselection. One can see a decrease in the amplitude of higher-order modes due to changes in the refractive index. Measurements presented in Figure 23b,d and Figure 24b,d were performed by immersing TFBG (b) and P-TFBG (d) sensors in aqueous solutions of glucose with a refractive index equal to 1.3471.

Figure 23 summarizes the most significant results from the application point of view. Figure 23a shows the spectral characteristics for the three polarizations P, S, and S|P for the TFBG structure placed in air. Analogous characteristics except for the TFBG structure placed in a glucose aqueous solution were measured in Figure 23b. Figure 23c shows the results of the spectral measurements for the P-TFBG structure in air, while Figure 23d shows the results of analogous spectral measurements for the P-TFBG system placed in a glucose aqueous solution. Significant changes and sensitivity of the spectral characteristics to the change in polarization can be seen. Comparing Figure 23a with Figure 23c and Figure 23b with Figure 23d, there is a clear reduction in the sensitivity of P-TFBG structures to changes in the polarization of the input light. After minimizing the effect of changes in this polarization, we performed refractive index measurements. The change in RI corresponded to changes that are induced, for example, by pathogens in the form of viruses. However, this required the development of a method that eliminates the sensitivity of the sensor signal to factors other than refractive index.

As shown in Section 3.3, the strongest dependence of the transmission coefficient for the central wavelength of the cladding mode was characterized by the structure with P-TFBG 7, in which the cladding modes @1539.8 nm and @1541.05 nm had the highest sensitivity to changes in RI (Figure 20 and Figure 21).

Spectral measurements of the P-TFBG characteristics were performed for a number of different solutions differing in RI value. Figure 24 shows the measurement system including the schematic diagram of the system and the stand with prepared glucose solutions with a strictly defined refractive index.

A change in the refractive index in the range from 1.344015 to 1.34672 caused noticeable and measurable changes in the spectral characteristics of P-TFBG structures. Transmission spectra were measured for SRI obtained with glucose solutions, which were vaporizing and constantly weighed on an electronic scale. To avoid any heterogeneity of concentration, the analytes were stirred. Table 2 contains a concentration of glucose and the corresponding refractive indices of analytes in which the P-TFBG structure was immersed.

**Figure 25:**
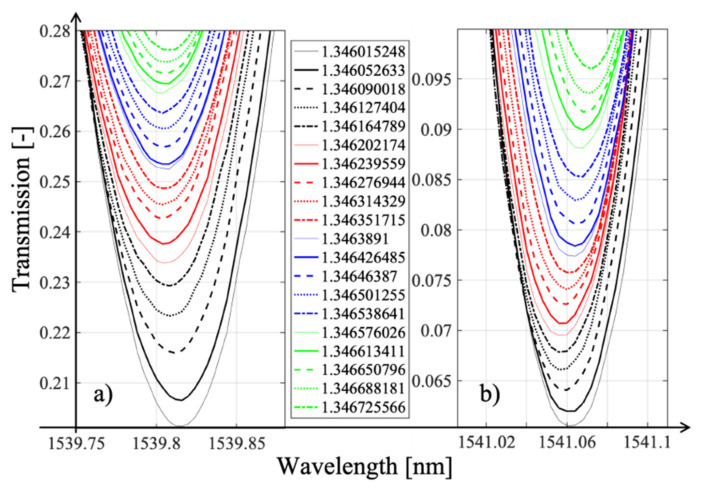
Refractive index measurement results for selected P-TFBG cladding modes: (**a**) @1539.8 nm, (**b**) @1541.05 nm.

The transmission coefficient of a particular mode was changed, causing a change in the height of the peak on the transmission spectral characteristic corresponding to this mode. Note also that the wavelength corresponding to the minimum value of the transmission coefficient for a given mode was also slightly changed (Figure 25). For measurements of very small changes in the refractive index, however, measurements of the minimum spectral characteristic should be recommended. The shift in this minimum can also be caused by a change in temperature.

In principle, a change in temperature does not cause a change in the transmission coefficient for individual modes. As a result of its changes, the whole TFBG spectrum is shifted, and consequently, P-TFBG is also shifted.

**Figure 26:**
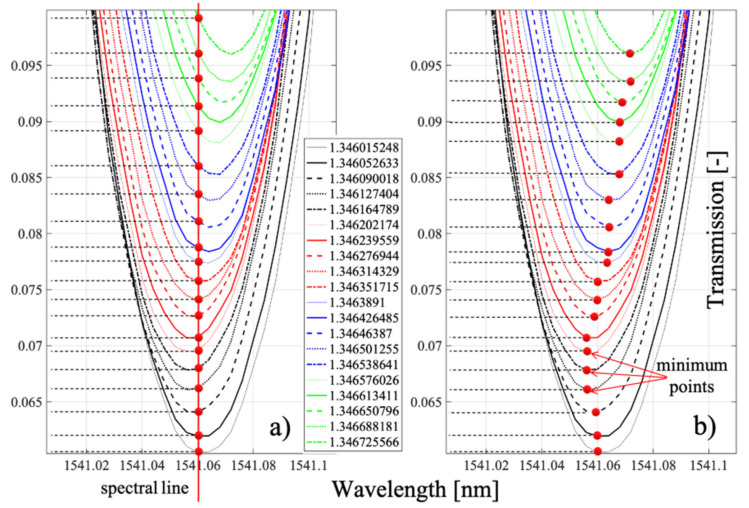
Graphical representation of how to determine refractive index changes using the P-TFBG structure: (**a**) spectral line method, (**b**) minimum tracking method.

Figure 26 illustrates the two discussed methods of performing indirect refractive index measurements. Mode @1541.05 nm was chosen for the analysis. We called this the spectral line method and the method of tracking the minimum on a spectral characteristic and determining the transmission coefficient corresponding to the minimum on such a characteristic.

The values of the transmission coefficient corresponding to varying values of the refractive index were different for the two methods. However, this is not very important because for both methods, the measurement is unambiguous, and the sensor and the whole measurement system can be calibrated in such a way that both the spectral line method and the minimum tracking method can be measured correctly.

**Figure 27:**
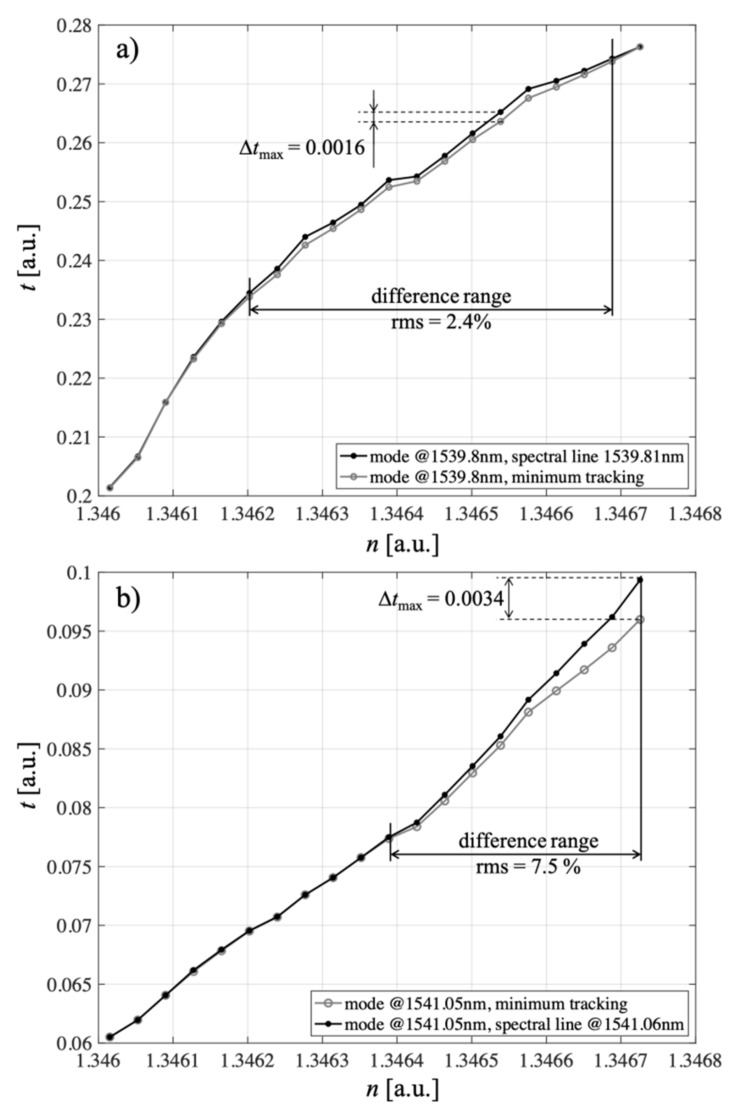
Processing characteristics for refractive index *n* measurements for two selected P-TFBG cladding modes: (**a**) @1539.8 nm, (**b**) @1541.05 nm. Measurements were performed using two methods: spectral line and minimum tracking.

To investigate the sensitivity of the proposed sensor to changes in refractive index, we present the processing characteristics for the selected two modes in Figure 27, marking the values of the sensitivity coefficient on them. In this paper, we proposed two methods for determining the refractive index, which were not much different from each other but important in the subsequent implementation. The RI can be determined from changes in the value of the transmission coefficient for a given single (fixed) wavelength. The study was carried out for two selected cladding modes of the P-TFBG structure @1539.8 nm and @1541.05 nm. As seen, the choice of the indirect measurement method affected the measured parameter of the spectrum (i.e., the value of the transmission coefficient of the structure0, but on the other hand, it can be seen that both in the case of the spectral line method and the minimum tracking method, the processing characteristics of the sensor were monotonic. They provide the possibility to unambiguously determine the measured refractive index. Characteristically, when the minimum tracking method is used, the processing characteristics are nearly linear, which is a desirable feature for many measurement systems. The maximum difference in transmission coefficient measurement for both methods was *t*_max_ = 0.0016 in the case of mode *n* containing from and 0.0034 in the case of mode @1541.05 nm, which did not seem to be a significant quantity, especially when the range of transmission coefficient variations for mode @1539. 8 nm was in the range from 0.201 to 0.278, while in the case of the @1541.05 nm mode, it was in the range from 0.061 to 0.099. It is also characteristic that the choice of the mode of the P-TFBG structure determines the obtained differences in *t*-measurements by the two methods (spectral line and minimum tracking).

**Figure 28:**
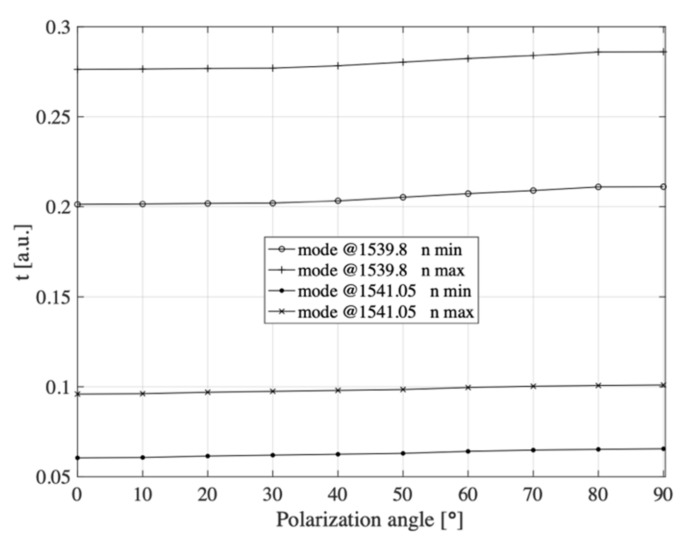
Characteristics of the cross-sensitivities of the P-TFBG sensor for changes in input light polarization angle *χ* measured for two selected cladding modes. Measurements were performed using the minimum tracking method.

For comparison, in Figure 28, we show the conversion characteristics of the input light polarization changes on the transmission coefficient of the same modes that were chosen for the RI change measurements. The measurements were made by varying the input light polarization angle from 0° to 90° using only the minimum tracking method. To denote the three specific polarization states, they were labeled S, S|P, and P in the paper, while in the case of tracking the exact changes of the polarization angle, we introduced the designation of this angle as *χ*. The labeled values *n* min and *n* max in Figure 28 correspond to the minimum and maximum measured values of the refractive index, respectively; in the paper, these values were 1.3461 and 1.3471, respectively.

By analyzing Figure 28, it can be seen that changing the polarization of the input light had very little effect on the transmission coefficient of the P-TFBG structure, as verified and reported earlier. Compared to the refractive index changes, this change was much smaller. It should also be noted that the nature of the transmission coefficient changes due to polarization changes is the same for several different refractive index values. The curves had the same characteristic of change and the same shape and slope. Regardless of the value of the refractive index being measured, the effect of polarization was the same.

To determine the metrological parameters, we determined the matrix equation of the P-TFBG sensor processing in the general form:

(6) $$\left[\begin{array}{c} P_{1}\\ P_{2} \end{array}\right]=\left[\begin{array}{cc} K_{n1} & K_{\chi 1}\\ K_{n2} & K_{\chi 2} \end{array}\right]\times \left[\begin{array}{c} n\\ \chi \end{array}\right]$$

where *K_n_*_1_ is the sensitivity of the parameter *P*_1_ to a change in the refractive index *n*; *K_n_*_2_ is the sensitivity of the parameter *P*_2_ to a change in the refractive index *n*; *K_χ_*_1_ is the sensitivity of the parameter *P*_1_ to a change in the input light polarization angle *χ*; and *K_χ_*_2_ is the sensitivity of the parameter *P*_2_ to a change in the input light polarization angle *χ*. Let us now explain what *P*_1_ and *P*_2_ mean in the case of our P-TFBG sensor. According to the information in this section, the determination of the refractive index is possible by measuring the transmission coefficient of selected cladding modes of the whole P-TFBG structure. According to the results presented in Figure 27 and Figure 28, the best measure of such changes is the value of the transmission coefficient of the selected cladding mode determined by two methods: the spectral line method and the minimum tracking method. However, since, according to the results presented in Section 3.2 and Section 3.3, the transmission coefficient was also affected by the change in the polarization angle of the input light, where this P-TFBG specificity obviously defines the parameters *P*_1_ and *P*_2_. Thus, the parameter *P*_1_ denotes the transmission coefficient determined by the spectral line method and is further denoted as *t_sl_*, while the parameter *P*_1_ denotes the transmission coefficient determined by the minimum tracking method and is further denoted as *t_mt_*.

Thus, after taking into account the values of the sensitivity coefficients determined experimentally, we are able to write the P-TFBG processing matrix Equation (6) for the @1539.8 nm mode in the following useful form:

(7) $$\left[\begin{array}{c} t_{sl}\\ t_{mt} \end{array}\right]=\left[\begin{array}{cc} 105.55 & 0.000110\left[1/{}^{\circ}\right]\\ 105.55 & 0.000111\left[1/{}^{\circ}\right] \end{array}\right]\times \left[\begin{array}{c} n\\ \chi \end{array}\right]$$

and in the case of the mode @1541.05 nm:

(8) $$\left[\begin{array}{c} t_{sl}\\ t_{mt} \end{array}\right]=\left[\begin{array}{cc} 49.96 & 0.000056\left[1/{}^{\circ}\right]\\ 54.70 & 0.000057\left[1/{}^{\circ}\right] \end{array}\right]\times \left[\begin{array}{c} n\\ \chi \end{array}\right]$$

As we can see, a 2-fold reduction in the sensitivity factor to *n* in the case of the @1541.05 nm mode also resulted in an approx. 2-fold decrease in the sensitivity to changes in *χ*.

When the condition of the nonzero determinant of the matrix is met, we can determine its complement matrix, and we will write the inverse processing equation in a form that allows indirect measurement of RI by measuring the transmission coefficient. In this way, an indirect measurement of the refractive index is possible using Equation (8). This equation also shows that the values of sensitivity to changes in polarization of the input light were negligibly small, and it is possible to indirectly measure the refractive index both by measuring the transmission coefficient for a given wavelength and its measurement by tracing the selected transmission minimum of the P-TFBG structure.

## 4. Future Works

The described system, sensor, and measuring method also have the potential to simultaneously measure temperature and refractive index or carry refractometric measurements insensitive to temperature changes. This is possible because a temperature change causes a shift in the entire spectral characteristics of the FBG structure [63,64] due to both the temperature expansion phenomenon and the so-called thermo-optical effect. Additionally, in the case of TFBG structures, a change in temperature produces the same effects and leads to a spectral shift of both the minima of the main Bragg resonance and the minima corresponding to the individual cladding modes [50]. Therefore, it is expected that, in principle, the P-TFBG structure will also be subject to the same phenomena. The sensitivity of the spectral shift to temperature can be described by the relation:

(9) $$K_{T}=\frac{\Delta \lambda }{\Delta T}=k_{T}\lambda_{\min}$$

where *λ* is the wavelength change; the spectral shift, *λ*_min_ is the wavelength corresponding to the minimum value of the transmission coefficient of the selected P-TFBG cladding mode; and *k_T_* is the relative temperature sensitivity coefficient equal to:

(10) $$k_{T}=\left(c_{\exp}+c_{to}\right)\frac{1}{K}=\left[\left(\frac{1}{\Lambda }\frac{\partial \Lambda }{\partial T}\right)+\left(\frac{1}{n_{eff}}\frac{n_{eff}}{\partial T}\right)\right]\frac{1}{K}$$

The parameter *c_exp_* is the temperature expansion coefficient of the optical fiber, and for quartz glass, its value is 0.55 × 10^−6^ K^−1^; *c_to_* is the thermo-optical coefficient, equal to 8.6 × 10^−6^ K^−1^.

In future work, we plan to perform thermal analyses of P-TFBG sensors. The proposed sensor could be used to measure both the refractive index in terms of detecting the presence of pathogens in blood and the temperature.

Of course, as we stated at the end of Section 3, the value of the transmission coefficient t can be determined by both the spectral line method (*t_sl_*) and the minimum tracking method (*t_mt_*). However, for practical purposes, it would be more convenient to use the value of *t_mt_* because in the case of a change in temperature, the whole spectral characteristic of the P-TFBG structure shifts, and tracking the shift of a selected minimum on this characteristic is more convenient and technologically simpler to realize.

## 5. Conclusions

In this paper, we demonstrated the possibility of measuring refractive index changes at a high level. To our knowledge, this is the first time in the literature that we have proposed the use of perpendicular TFBG P-TFBGs for RI detection. In this paper, we demonstrate the possibility of theoretically determining the transmission coefficient of the TTFBG structure for each polarization state represented by the angle of linearly polarized light. It has been shown that the effect of changes in the polarization angle of the input light in the range from 0° to 90° on the transmission spectra is significantly reduced when the twist angle of such a structure increases. It has been demonstrated that the twist occurring in a TTFBG structure causes a decrease in its sensitivity to the polarization of the input light.

It was also shown that the nature of the transmission coefficient changes due to polarization changes was the same for several different values of the measured refractive index. The curves had the same characteristic of changes and the same shape and slope. Regardless of the value of the measured refractive index, the effect of polarization was the same. This makes it possible to calibrate the sensor and make precise refractometric measurements.

## Figures and Tables

**Table 1:** Parameters of the produced TTFBG structures.

	*α* = 180°		α = 90°		*α* = 45°		*α* = 0°	
*T* [-]	min	max	min	max	min	max	min	max
	0.720	0.740	0.635	0.824	0.635	0.824	0.546	0.895
*T* [-]	0.0200		0.0900		0.1894		0.3493	
δ*T* [%]	2.78		11.54		23.76		39.03	
K^*T*^_α_ [1/°]	0.0002		0.0010		0.0021		0.0039	
*T*_|0°_	0.72		0.78		0.7972		0.895	

**Table 2:** The refractive index values of the analyte used.

Glucose Concentration	Refractive Index	Glucose Concentration	Refractive Index
[%]	[RIU]	[%]	[RIU]
8.810160	1.346015248	9.059395	1.34638910
8.835083	1.346052633	9.084318	1.346426485
8.860007	1.346090018	9.109241	1.346463870
8.884930	1.346127404	9.134165	1.346501255
8.909854	1.346164789	9.159088	1.346538641
8.934777	1.346202174	9.184012	1.346576026
8.959701	1.346239559	9.208935	1.346613411
8.984624	1.346276944	9.233859	1.346650796
9.009548	1.346314329	9.258782	1.346688181
9.034471	1.346351715	9.283706	1.346725566

## Data Availability

Not applicable.
